# Personality traits and stress coping among obstetricians diagnosing and communicating fetal death: A cross‐sectional study

**DOI:** 10.1002/ijgo.14048

**Published:** 2021-12-11

**Authors:** Dana Anais Muin, Janina Erlacher, Stephanie Leutgeb, Bettina Toth, Anna Felnhofer

**Affiliations:** ^1^ Division of Fetomaternal Medicine Department of Obstetrics and Gynecology Comprehensive Center for Pediatrics Medical University of Vienna Vienna Austria; ^2^ Austrian Society of Obstetrics and Gynaecology (OEGGG) Vienna Austria; ^3^ Gynecological Endocrinology and Reproductive Medicine Medical University Innsbruck Innsbruck Austria; ^4^ Division of Pediatric Pulmonology, Allergology and Endocrinology Department of Pediatrics and Adolescent Medicine Comprehensive Center for Pediatrics Medical University of Vienna Vienna Austria

**Keywords:** empathy, fetal death, gender, locus of control, stillbirth, stress coping

## Abstract

**Objective:**

To assess obstetricians’ personality traits (empathy, locus of control [LoC], situational affect) and relate these to stress coping when making the diagnosis and delivering the news of late fetal death to parents.

**Methods:**

Cross‐sectional questionnaire study.

**Results:**

341 Austrian obstetricians (72.7% females) participated in this online survey. Participants’ mean age was 46.4 ± 10.8 years. The majority of participants (*n *= 158, 46.3%) had been previously involved in the diagnosis of fetal death and subsequent breaking news up to five times. We observed no gender‐specific differences in physicians’ stress coping, including situational affect, perceived stress, challenge, self‐concept, or perceived control, nor in internal or external LoC, and perspective taking. Female obstetricians showed significantly higher trait empathy and reported higher levels of distress regarding fetal death than males. Obstetricians with greater experience in dealing with fetal death (>11 times) reported a higher ability to cope with stress as reflected by lower situational affect, less perceived stress, less challenge, and higher situational control.

**Conclusion:**

While obstetricians’ stress coping in diagnosing and communicating fetal death is independent of physicians’ gender, greater ability to empathize with the parents diminishes overall sense of control and affect over the situation, whereas increased level of clinical experience with fetal death supports all domains of control and stress coping.

## Abstract 2

SynopsisThe contents of this page will be used as part of issue TOC only. It will not be published as part of main article.

Synopsis

Stress coping in diagnosing and communicating fetal death is independent of obstetricians’ gender, yet clinical experience increases, while empathy diminishes, situational control and affect.

## 1 INTRODUCTION

Communicating adverse pregnancy outcome, such as fetal death, is a very delicate situation that requires empathy and self‐reflective behavioral skills towards the affected individuals. At the same time, diagnosing and communicating fetal death evokes a moderate to high degree of stress among obstetricians and requires internally‐ and externally‐mediated strategies to handle this situation appropriately and to gain professional resilience.^1^

Breaking bad news in medicine, in general, has been repeatedly found to distress and overwhelm physicians across all disciplines.^2^, ^3^ According to the *transactional model of stress and coping*, stress occurs as a result of an imbalance between external demands and internal (personal) resources.^4^ The individual's coping reaction, in turn, involves a range of cognitive, emotional and behavioral responses, which are directed towards managing the stressors. Likewise, empathy has received attention in the context of dealing with stressful clinical practice.^5^ While empathic concern for others is thought to constitute a prerequisite for a satisfactory patient‐physician relationship,^6^ too much empathic involvement may also lead to higher levels of distress when communicating bad outcomes to patients.^5^

Similar to empathy, the extent to which a person has the impression to have control over a situation may impact stress coping. The personality trait “locus of control” (LoC) encompasses one of two opposing convictions, i.e., the belief that one's actions have an influence on events (internal LoC), and the belief that events are influenced by external sources, such as other persons or fate (external LoC).^7^ Past studies have confirmed stress coping differences between individuals with internal versus external LoC: Persons with an internal LoC tend to perceive less stress and have better coping skills, while externals provoke more negative moods.^8^, ^9^

Lastly, prior work experience may also have an impact on stress coping and empathic concern.^5^ Those who have previously experienced and managed difficult situations successfully are considered better equipped to cope with comparable stressors than novices. Similarly, gender has been linked to stress coping, with differences in stress reactivity and coping styles between males and females, and to impact empathy, with females showing higher empathy levels than males.^10^, ^11^

The paucity of evidence on the influence of these qualities on physicians’ perspective taking with stillbirth justified the attempted in‐depth identification and characterization of co‐dependent factors in handling this situation. Our research was guided by the rationale of wanting to better understand why some physicians cope better with fetal death than others. With this study, we therefore sought to assess obstetricians’ personality traits (i.e., empathy, LoC) and relate them to stress coping when diagnosing and breaking the news of late fetal death to parents. We furthermore aimed to explore the role of obstetricians’ gender and level of experience on stress coping under these special circumstances.

## 2 METHODS

### 2.1 Overview of antenatal care in Austria

In Austria, where this study was conducted, all publicly insured women are entitled to thorough and consistent antenatal care from the onset of pregnancy until delivery. In 1974, the Austrian Government introduced a stamp collection system (“*Mother*‐*Child*‐*Booklet*”) entailing five obligatory check‐ups to ensure that all relevant maternal and fetal examinations are carried out and the pregnancy is objectively and closely monitored.^12^ For routine antenatal care, including the record keeping of the Mother‐Child‐Booklet, women attend private or public doctor's offices led by obstetricians and gynecologists outside hospital. In case of an emergency or any obstetric pathology, women are transferred to maternity units in hospitals for further management.

In Austria, intrauterine fetal death is usually confirmed by two independent ultrasonography scans, conducted either first by the specialist doctor at their office, followed by a second opinion at the hospital, or directly by two obstetricians (usually a junior and senior physician) at the hospital. As part of the training, residents may be present to witness and learn from the lead on how to convey the news of fetal death to the parents. Oftentimes, however, physicians are left on their own to handle the diagnoses and prepare the parents for the bad news.

### 2.2 Data collection

We conducted a cross‐sectional web‐based survey of 1526 obstetricians and gynecologists registered with the Austrian Society of Obstetrics and Gynecology (*Oesterreichische Gesellschaft fuer Gynaekologie und Geburtshilfe*; OEGGG). The questionnaire was conceived by the study team (DAM, JE, BT, AF) and approved by the Medical Board of the OEGGG as well as the Ethics Committee of the Medical University of Vienna. According to the Declaration of Helsinki, consent was obtained from all participants upon enrolling.

The survey link was created via SurveyMonkey (https://www.surveymonkey.de/), an online tool which allows collecting data anonymously without storing sensitive background information (i.e., IP address). No cookies were used in this survey. The survey was closed and protected against unauthorized access. Access to the survey data source and email list was restricted to one investigator (SL).

The online questionnaire was checked regarding usability and technical functionality. An oral interview was conducted with five voluntary participants upon filling in the questionnaire to assess the reliability of the survey, and yielded a minimal variance of 0.1% to the written responses.

An invitation email to this survey was distributed to all Austrian obstetricians and gynecologists via the OEGGG email server between September and October 2020 with two subsequent reminders by weeks 2 and 3. Participation was by free choice with no incentives. Participants learned about the purpose of the study, the average length of the survey (15 min), the investigator team and the anonymity of their data. Participants were also informed that the results would be published and presented to the public. All responses were anonymous and no direct personal information was stored. Duplicate entries were avoided by preventing users access to the survey twice. Only completed questionnaires were analyzed and data were transferred into an excel file sheet and checked for integrity and consistency.

### 2.3 Measures

The questionnaire consisted of 16 items and included three validated questionnaires to collect demographic data, experience, trait empathy,^13^, ^14^ locus of control,^7^, ^15^, ^16^ stress coping,^17^, ^18^ and affect in the context of late singleton intrauterine fetal death (≥20 gestational weeks).

#### 2.3.1 Demographics

Upon enrollment, participants were asked to share their demographic characteristics regarding *gender* (male; female; others), *current position* (residency; specialist doctor in public or private practice or hospital; consultant; departmental head; retired), *relationship status* (single; liaised; married; divorced; widowed), *age* (in years), and *number of children* (*n*).

#### 2.3.2 Experience

Clinical experience in diagnosing and/or delivering the news of fetal death to affected parents was categorized into four groups according to the frequency of previous cases (*“0”*, *“*<*5”*, *“6*–*10”*, *“*≥*11” times)*. Participants with no prior experience in diagnosing and/or communicating fetal death (“0 times”) were excluded from this analysis.

### 2.4 Empathy

The validated German version of the Interpersonal Reactivity Index,^13^ the *Saarbrücker Persönlichkeits*‐*Fragebogen (SPF)*,^14^ was applied to measure the psychological trait empathy. For the current study, the following three (out of originally four) subscales containing 12 items on a 5‐point Likert scale (1 = does not apply, 5 = fully applies) were used: (i) perspective taking (describes the ability to adopt another person's point of view; e.g., “*In a dispute*, *I try to understand both sides before I make a decision*”), (ii) affective empathy (i.e., the tendency to be affected by another's emotional state; e.g.,”*When I see someone being exploited*, *I feel the need to protect them*”), and (iii) distress (feelings of discomfort and anxiety in response to a person's suffering, e.g., “*In emergency situations*, *I feel anxious and uncomfortable*”). The fantasy scale was omitted in order to meet feasibility and time economy for the assessors, and to ensure comparability with previous literature.^5^

### 2.5 Locus of control

Based on the approach of Jakoby and Jacob (1999),^15^ who assumed a two‐factor solution to the construct LoC,^7^ the German self‐report questionnaire *Internale*‐*Externale*‐*Kontrollüberzeugung*‐*4* (*IE*‐*4*)^16^ was used. It consists of four items on a 5‐point Likert scale (1 = does not apply, 5 = fully applies), which may be summed up into the two subscales (i) *Internal LoC* (i.e., reflecting a person's belief of being in the control of their own destiny), and (ii) *External LoC* (i.e., reflecting the belief that one's fate is determined by luck and powerful others).

#### 2.5.1 Stress coping

Participants’ coping with stress at breaking news of fetal death was assessed by individual rating of the following four items on a 5‐point Likert scale (1 = does not apply, 5 = fully applies)^17^, ^18^, ^19^: (i) *“The situation was stressful for me”* (perceived stress), (ii) “*I found the situation to be a challenge*” (challenge), (iii) *“I knew what I had to do to influence the situation*” (self‐concept), and (iv) “*I was able to do something to influence the course of the previous situation”* (perceived control).

### 2.6 Affect

Finally, the situational affect during breaking news of fetal death was rated by the participants on a 5‐point Likert scale (“*How did you feel?*”) (1 = very bad, 5 = very good).

### 2.7 Statistical analyses

The distribution of data was analyzed using the Kolmogorov–Smirnov test, normally distributed metric data are presented as mean and standard deviation (M ± SD). Categorical data are given as absolute (*n*) and relative frequencies (%). Continuous data were compared with Kruskal–Wallis test with Dunn's multiple comparison test. Univariate Analyses of Variance (ANOVAs) with between‐factors gender (male/female) and experience (i.e., number of delivered diagnoses; 3 groups: *“*<*5”*, *“6*–*10”*, *“*>*11”*) were used to analyze participants’ self‐reported stress coping and affect when delivering the diagnosis of fetal death. Post‐hoc tests (Tukey, 95% Confidence interval; CI) were reported for all comparisons. Median splits were calculated for trait empathy (SPF subscales) and LoC (IE4 subscales), and subsequent group comparisons between high and low trait empathy and LoC, respectively, were undertaken by the means of student t‐tests. In addition, Chi^2^‐tests (χ*^2^*) were used for categorical variables. All reported *P*‐values are two‐sided, and a *P*‐value of <0.05 was considered significant with a 95% CI. Statistical tests were performed with SPSS Statistics Version 26 (IBM Corporation). Figures were created by GraphPad Prism 9 for macOS (GraphPad Software, LLC).

## 3 RESULTS

### 3.1 Sample characteristics

**FIGURE 1:**
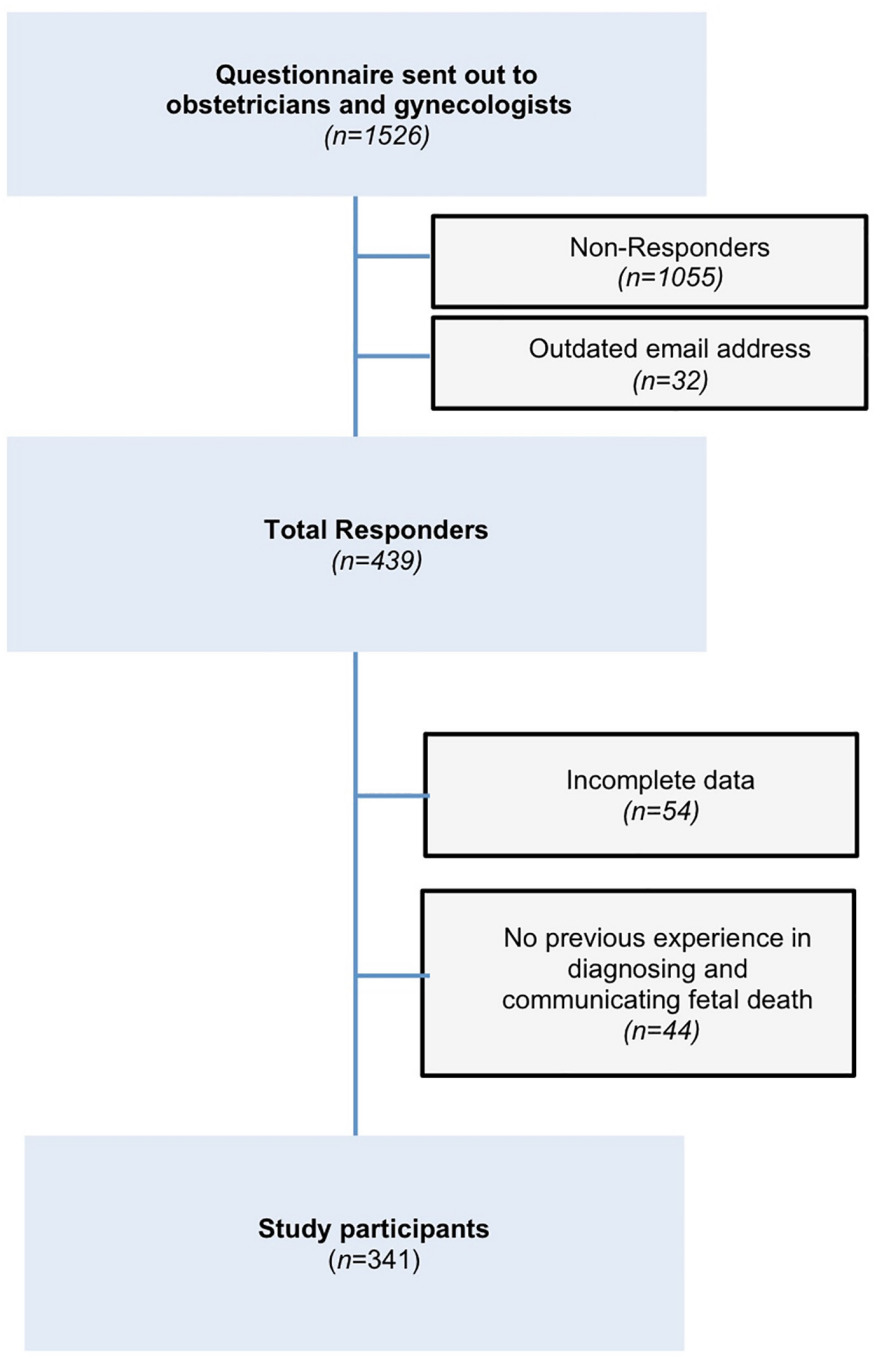
Flowchart illustrating the enrolment of participants in this study (*n *= 341) who anonymously replied to an online questionnaire conveyed by the Austrian Society of Obstetrics and Gynecology between 21 September 2020 and 31 October 2020

Initially, *n *= 439 participants enrolled in the online survey (Figure 1). Dropouts, incomplete data sets and those with conspicuous response patterns were excluded, as well as participants with no prior experience in diagnosing and/or communicating fetal death (*n *= 44). The final sample, therefore, constituted 341 obstetricians [*n *= 248 (72.7%) females; *n *= 93 (27.3%) males], whose baseline characteristics are shown in Table 1.

**TABLE 1:** Baseline characteristics of obstetricians who participated in the online survey conducted by the Austrian Society of Obstetrics and Gynecology between 21 September 2020 and 31 October 2020 (*n *= 341)

	Frequency of previous deliveries of diagnosis of fetal death			Group differences
	<5 (*n* = 158)	6–10 (*n* = 84)	>11 (*n* = 99)	
Female participants	84.2%	74.7%	52.5%	χ^2^(2) = 30.918 *P *< 0.001
Age (years; *M* ± SD)	41.99 ± 10.36	47.17 ± 9.26	52.96 ± 9.26	*F*(2,320) = 36.489 *P *< 0.001
Current position (*n*, %)				
Residency	49 (31.4%)	6 (7.2%)	2 (2.0%)	χ^2^(14) = 91.256 *P *< 0.001
Specialist doctor (public practice)	25 (16.0%)	19 (22.9%)	21 (21.2%)	
Specialist doctor (private practice)	25 (16.0%)	17 (20.5%)	13 (13.1%)	
Specialist doctor (hospital)	33 (21.2%)	12 (14.5%)	10 (10.1%)	
Consultant (hospital)	21 (13.5%)	27 (32.5%)	33 (33.3%)	
Departmental head	2 (1.3%)	2 (2.4%)	18 (18.2%)	
Retired	1 (0.6%)	0 (0.0%)	2 (2.0%)	
Relationship status (*n*, %)				
Single	14 (9.6%)	10 (12.3%)	12 (12.2%)	χ^2^(8) = 4.111 *P *= 0.847
Liaised	29 (19.9%)	13 (16.0%)	15 (15.3%)	
Married	96 (65.8%)	53 (65.4%)	66 (67.3%)	
Divorced	7 (4.8%)	5 (6.2%)	4 (4.1%)	
Widowed	0 (0.0%)	0 (0.0%)	1 (1.0%)	
Children (*n*, %)				
1	26 (27.7%)	14 (25.0%)	11 (26.4%)	χ^2^(8) = 9.749 *P *= 0.283
2	47 (50.0%)	23 (41.1%)	41 (61.2%)	
3	16 (17.0%)	15 (26.8%)	13 (19.4%)	
4	5 (5.3%)	3 (5.4%)	2 (3.0%)	
5	0 (0.0%)	1 (1.8%)	0 (0.0%)	

Note

*M* ± SD = mean ± standard deviation.

The study population's mean age was 46.4 ± 10.8 years (range 24–67 years) with a significant age difference between male and female doctors [53.63 ± 9.61 versus 43.80 ± 10.04 years; *t*(322) = −7.903, *P *< 0.001]. Most physicians indicated that they had delivered the diagnosis of fetal death up to five times (*n *= 158, 46.3%). Male physicians indicated that they had delivered the diagnosis more often than females [χ*^2^* (2) = 30.918, *P *< 0.001]. Obstetricians with the most experience in breaking news of fetal death (>11 times) were significantly older [*F*(2,320) = 36.489, *P *< 0.001].

#### 3.1.1 Gender‐specific differences in coping with stress

One‐way ANOVA did not reveal any gender differences with regards to stress coping, including situational affect [*F*(1,337) = 2.751, *P *= 0.098], perceived stress [*F*(1,339) = 2.323, *P *= 0.128], challenge [*F*(1,339) = 0.199, *P *= 0.656], self‐concept [*F*(1,339) = 1.363, *P *= 0.244], or perceived control [*F*(1,339) = 1.136, *P *= 0.287].

Similarly, male and female obstetricians did not differ in their internal LoC [*F*(1,317) = 3.398, *P *= 0.066] or external LoC [*F*(1,317) = 0.850, *P *= 0.357], nor in their perspective‐taking ability [*F*(1,317) = 0.268, *P *= 0.605]. Female obstetricians, however, showed significantly higher trait empathy than male obstetricians [15.39 ± 3.00 vs. 14.32 ± 2.72, respectively; *F*(1,317) = 8.209, *P *= 0.004], and females reported higher levels of trait distress than males [7.95 ± 2.65 vs. 6.77 ± 2.47, respectively; *F*(1,317) = 12.648, *P *< 0.001].

#### 3.1.2 Experience and coping with stress

**FIGURE 2:**
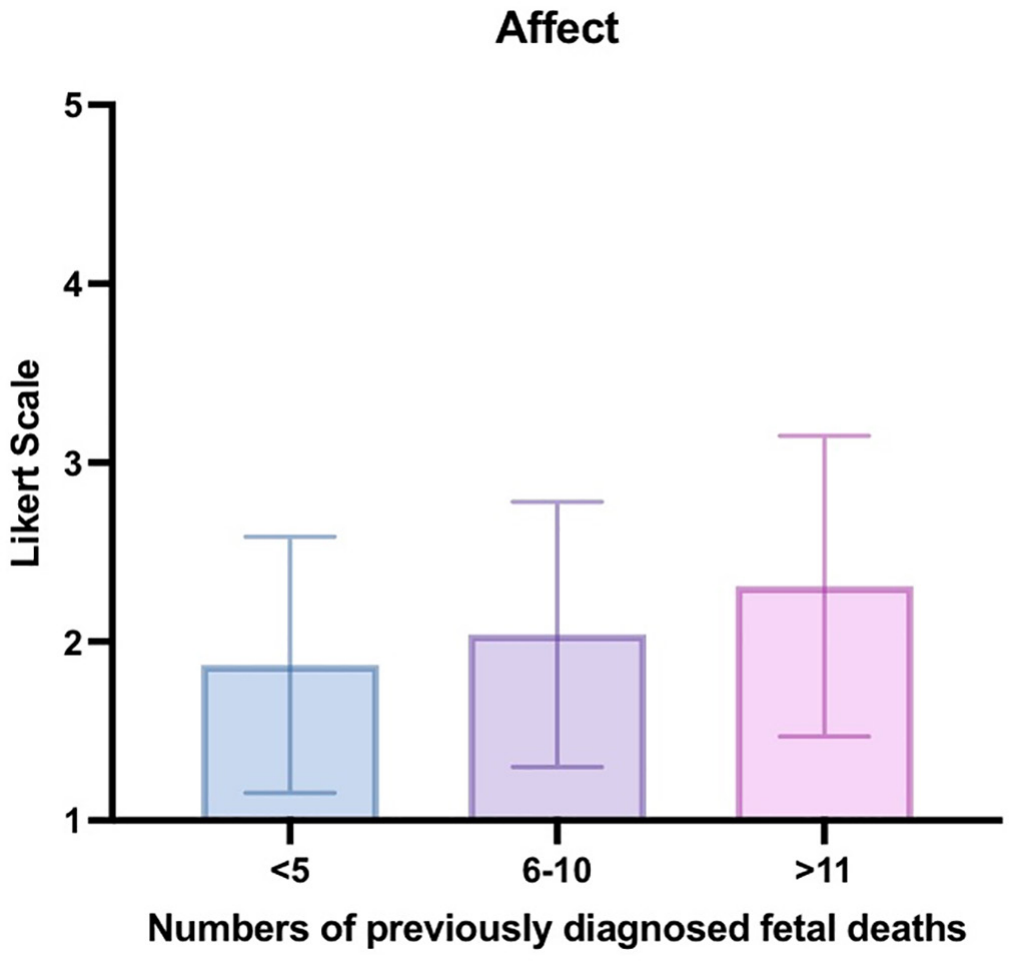
Interleaved bar chart with error bars on mean and standard deviation illustrating obstetricians’ affect (“How did you feel”; *n *= 341) as assessed on a 5‐point Likert Scale (1 = very bad, 5 = very good) per level of experience in having diagnosed and delivered the news of fetal death to affected parents (i.e., never 0; <5 times; 6–10 times; ≥11 times)

In comparison between levels of clinical experience in breaking news of fetal death (<5 times; 6–10 times; >11 times), we found significant group differences regarding situational affect [*F*(2,335) = 10.208, *P *< 0.001], with Tukey post‐hoc analyses showing less negative affect for the most experienced cohort (>11 cases), than for the least experienced cohort [0.441 (0.21, 0.67), *P *< 0.001; Figure 2].

**FIGURE 3:**
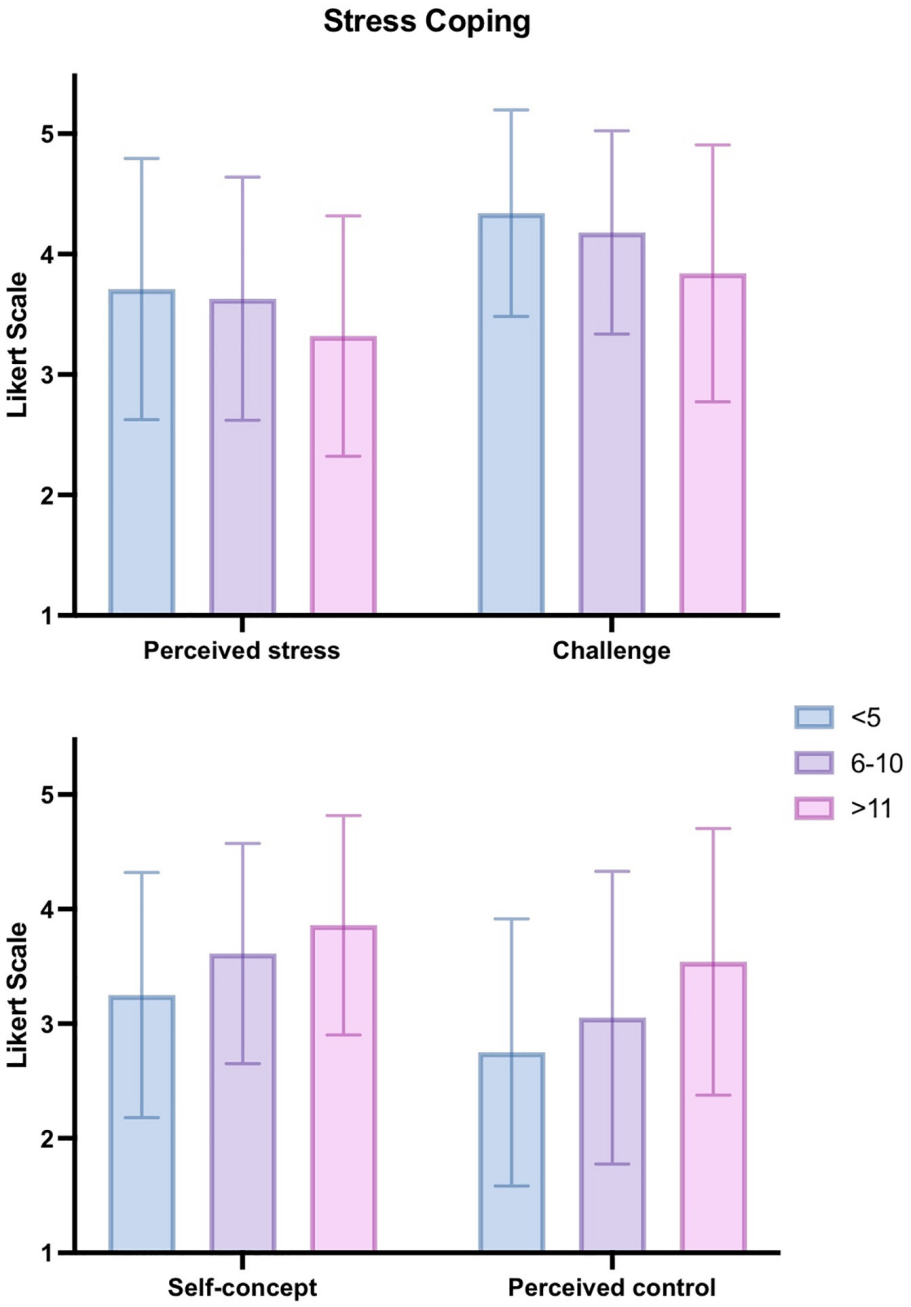
Interleaved bar chart with error bars on mean and standard deviation illustrating obstetricians’ stress coping comprising four domains (Perceived stress: “The situation was stressful for me”; Challenge: “I found the situation to be a challenge”; Self‐concept: “I knew what I had to do to influence the situation”; Perceived control: “I was able to do something to influence the course of the previous situation”; *n *= 341) as assessed on a 5‐point Likert Scale (1 = does not apply, 5 = fully applies) and per level of experience in having diagnosed and delivered the news of fetal death to affected parents (i.e., never 0; <5 times; 6–10 times; ≥11 times)

Figure 3 illustrates the influence of clinical experience with fetal death on all four domains of stress coping (i.e., perceived stress; challenge; self‐concept; perceived control): Tukey (*P *= 0.011) revealed less perceived stress for the most experienced group when compared to the least experienced one [0.386 (0.07,0.70); *F*(2,337) = 4.297, *P *= 0.014]. Similarly, the most experienced group found the situation to be less of a challenge than the group with little experience in delivering the news of fetal death [<5 times; Tukey: *P *< 0.001; 0.503 (0.23,0.78); *F*(2,337) = 9.151, *P *< 0.001].

The least experienced group had the least resources to draw from to influence the situation in contrast to the more [Tukey: *P *= 0.021; −0.368 (−0.69, −0.04)], and most experienced groups [Tukey: *P *< 0.001; −0.612 (−0.92, −0.31); *F*(2,337) = 11.656, *P *< 0.001]. Finally, obstetricians with the highest clinical experience (>11 cases) had a significantly higher perceived control over the situation than those with 6–10 previous cases [Tukey: *P *< 0.001; 0.487 (0.07,0.91)] and than the least experienced [Tukey: *P *< 0.001; −0.782 (1.14,0.42); *F*(2,337) = 13.078, *P *< 0.001].

#### 3.1.3 Empathy, locus of control and coping with stress

Student t‐tests revealed no significant group differences between the external and internal LoC groups or between those scoring high and low on perspective taking with regards to stress coping and affect (Table 2).

**TABLE 2:** Means (M) and standard deviations (±SD) for median split groups of Locus of Control (LoC) and psychological trait empathy (i.e., perspective taking, empathy, distress) as assessed by *Primary Appraisal Secondary Appraisal scale (PASA)* by Gaab et al.^19^ regarding stress coping and affect in obstetricians at delivering bad news of fetal death to affected parents

	Internal LoC		External LoC		Perspective taking		Empathy		Distress	
	High (*n* = 176)	Low (*n* = 143)	High (*n* = 111)	Low (*n* = 208)	High (*n* = 180)	Low (*n* = 139)	High (*n* = 201)	Low (*n* = 117)	High (*n* = 168)	Low (*n* = 151)
AFFECT “How did you feel?”	2.10 ± 0.87	1.97 ± 0.70	2.06 ± 0.72	2.03 ± 0.83	2.03 ± 0.76	2.06 ± 0.84	1.97 ± 0.82	2.16 ± 0.73	1.93 ± 0.72	2.14 ± 0.84
PASA 1 Perceived stress	3.59 ± 1.06	3.57 ± 1.07	3.53 ± 1.03	3.61 ± 1.08	3.60 ± 1.02	3.55 ± 1.12	3.71 ± 1.07	3.36 ± 1.01	3.71 ± 1.00	3.46 ± 1.11
PASA 2 Challenge	4.15 ± 1.03	4.17 ± 0.83	4.05 ± 0.90	4.22 ± 0.96	4.19 ± 0.96	4.12 ± 0.92	4.31 ± 0.86	3.91 ± 1.03	4.28 ± 0.82	4.05 ± 1.03
PASA 3 Self‐concept	3.59 ± 1.05	3.43 ± 1.01	3.39 ± 1.09	3.59 ± 1.00	3.55 ± 1.00	3.47 ± 1.09	3.52 ± 1.08	3.50 ± 0.95	3.31 ± 1.00	3.70 ± 1.04
PASA 4 Perceived control	3.11 ± 1.26	3.01 ± 1.20	2.95 ± 1.24	3.13 ± 1.23	3.14 ± 1.23	2.96 ± 1.24	3.05 ± 1.26	3.09 ± 1.19	2.82 ± 1.14	3.29 ± 1.27

Note

*M* ± SD = mean ± standard deviation.

Yet those who scored high on trait empathy felt significantly worse when delivering the diagnosis than those with lower empathy scores [*t*(315) = 2.107, *P *= 0.036]. Also, those high in empathy reported to have perceived the situation as more stressful [*t*(317) = −2.856, *P *= 0.005] and more challenging [*t*(317) = −3.747, *P *< 0.001] than those with low trait empathy.

At the same time, obstetricians with both high and low empathy skills showed neither significant differences in their self‐concept, thus knew what to do during the clinical encounter, nor in their sense of perceived control, thus were able to influence their course of medical and professional action.

Lastly, those who scored higher on trait distress reported feeling worse during verbal delivery of the diagnosis [*t*(314.257) = 2.328, *P *= 0.021]. In addition, they perceived the situation as significantly more stressful [*t*(317) = −2.059, *P *= 0.040] and as more of a challenge [*t*(317) = −2.265, *P *= 0.024], and were less oriented about what to do [self‐concept: *t*(317) = 3.432, *P *= 0.001; perceived control: *t*(317) = 3.415, *P *= 0.001].

## 4 DISCUSSION

### 4.1 Principal findings

Starting from the observation that breaking bad news may have severe effects on medical professionals across all disciplines,^2^, ^3^, ^20^, ^21^ we set out to identify factors in obstetricians which may—positively or negatively—be associated with their individual stress coping when encountering fetal death in their clinical practice.

Whilst female obstetricians yielded higher scores in empathy and distress when diagnosing and communicating fetal death, our study showed no gender differences with regards to stress coping, including situational affect, perceived stress, challenge, self‐concept, perceived control, nor internal and external locus of control.

The influence of level of experience on coping with stress in the circumstance of stillbirth clearly revealed that obstetricians with wider clinical experience reported less negative situational affect, stress or challenge, yet higher perceived situational control and stronger self‐concept.

Likewise, the effect of empathy on coping with stress in the circumstance of stillbirth was that physicians with stronger empathy reported higher situational affect, more perceived stress and challenge, yet remained constant in their perception of self‐concept and control over the situation.

Obstetricians with greater levels of trait distress revealed higher situational affect, more perceived stress and challenge, but reported poorer self‐concept and less situational control.

There were no differences between male and female obstetricians, neither in their self‐reported stress coping, nor in their situational affect when diagnosing fetal death. In other words, male and female physicians indicated equally experience high levels of stress and negative feelings in this challenging situation.

### 4.2 Interpretation

Our findings are supported by prior research, which found that physicians’ gender did not affect coping styles in clinically difficult situations.^2^, ^22^ In contrast to self‐reported stress levels, however, we did observe gender differences with regards to trait empathy in our sample: Female obstetricians scored higher on affective empathy and distress, meaning that they are generally more affected by the female patient's emotional state and feel more discomfort in response to a woman's suffering than their male colleagues. Quite consistently, women have been found to be more empathetic than males in past research,^23^, ^24^ hence, our findings fit in with the evidence. Given that female obstetricians reported being more empathetic, but did not indicate that they had experienced more stress or burden when confronted with fetal death, implies that they may use more effective coping mechanisms to deal with the situation. Additional research is warranted to more comprehensively explore the particular role obstetricians’ gender may play in coping with diagnosing fetal death.

Furthermore, we found differences between those obstetricians scoring high on the empathy scales and those scoring low, regardless of gender. Thus, obstetricians with high trait empathy and high distress reported having experienced higher levels of stress when communicating the diagnosis of fetal death. Also, they perceived the situation as more challenging and indicated having had less control over it. This is in line with the assumption that more empathy is associated with more personal distress in clinical care.^5^ Hence, those who are more emotionally involved in their patients’ suffering are also thought to be more impacted by emotionally laden situations. In the worst case, this may end up in compassion fatigue and burnout.^25^ Thus, it is essential to provide adequate support to those who tend to be highly empathetic in order to prevent long‐term burden as well as negative effects on daily decision making within the delivery room.

In contrast to empathy, there was a relation between LoC and stress coping. Obstetricians with an external LoC (i.e., the belief that events were influenced by external factors) reported comparable stress levels to those obstetricians who held the belief that they were the masters of their fate (internal LoC). In contrast to past studies in the general population,^8^, ^9^ the perception that one is able to manage the stress is not associated with LoC in obstetricians. This may be indicative of other personality traits, which may be more predictive of stress coping than LoC. For instance, Kwarta et al. (2016) have found neuroticism, extroversion and conscientiousness to be correlated with emotion‐oriented or task‐oriented coping styles in physicians.^22^ Thus, future studies should make an effort to include these variables in their analyses.

Finally, we found prior experience with fetal death to be linked to better stress coping in obstetricians. Those who indicated having diagnosed and communicated stillbirth more frequently (>11 times) were also less likely to experience stress and negative affect. Also, they reported being more in control of the situation and perceiving it less as a challenge. This finding is mostly in line with prior research showing that while senior and junior physicians perceived similar stress levels, the more experienced physicians tended to engage in more adaptive problem‐focused coping strategies.^3^ While being intuitive, this finding also highlights the need for extensive training and support of those with no or little work experience to better prepare them for these challenging clinical situations. On the one hand, it is critical that all staff dealing with perinatal death have a sound understanding of the clinical management and support for bereaved parents encountering stillbirth. On the other hand, it has been shown that health care providers may enhance their self‐confidence and medical conduct through training and skills sessions, such as the *IMproving Perinatal Mortality Review and Outcomes Via Education* tool.^26^

### 4.3 Strengths and limitations

Our study is not devoid of limitations inherent to its cross‐sectional study design as an online survey to assess the variables of interest. We therefore cannot assume representativeness of our sample, and we cannot validate the identity of those who participated. Also, we have no demographic characteristics available on those invitees who chose not to participate to rule out a participation bias among those who responded to the survey. Furthermore, we asked the participants to describe their stress reaction to stillbirth in retrospect. Recalling a situation is prone to recall bias, thus, a different method of assessment (e.g., conducting an interview closely after encountering fetal death) may have yielded different results. Lastly, we did not assess how many participants had formally received training in breaking news of fetal death.

Despite these limitations, our study focuses on the hitherto under‐researched subject of obstetricians’ reactions to and coping with diagnosing and communicating fetal death. The results provide valuable insights into how obstetricians handle these delicate situations and how prior experience and their trait empathy shape stress coping, which may be used as a starting point for further research. Also, our study considered a range of factors, such as demographic variables (i.e., gender, age), personality factors (i.e., LoC, trait empathy) and prior work experience, which are linked to coping strategies. Finally, we assessed the reliability of the constructed survey tool by an oral interview, which showed a very small variance.

## 5 CONCLUSION

Within the frame of diagnosing and communicating fetal death, situational affect, perceived stress and control, experience of challenge and self‐concept seem to be independent of physician's gender; however, females may display higher empathy scores, thus experiencing greater distress, than males. The ability to overcome the emotional burden of stillbirth seems to be positively influenced by physicians’ level of clinical experience.

## CONFLICT OF INTEREST

None declared.

## AUTHOR CONTRIBUTIONS

We confirm that all authors fulfilled all conditions required for authorship, as all authors contributed to the conception, planning and carrying out of the research. DAM conceived the study and wrote the first draft of this paper. DAM, JE, SL, BT and AF designed the study and questionnaire and helped with acquisition of data. DAM, SL and AF conducted statistical analyses. All authors contributed to critically revising the paper for important intellectual content and approved the final version to be published. All authors accept responsibility for the article as published.
